# St. Thomas's Hospital

**Published:** 1901-06-29

**Authors:** 


### ST. THOMAS'S HOSPITAL.

### OPENING OF NEW CHILDREN'S WARD AND OPERATING THEATRES.

Extensive alterations and additions are in progress at St. Thomas's Hospital, and Lord Lister presided on June 20 over the opening of a children's ward and operating theatres —a first instalment of the improvements in course of construction. St. Thomas's is always an ideal building for a ceremony, and many guests gathered in the entrance hall to hear Lord Lister's address, and to take part in the short form of Dedication Service read by the Bishop of Southwark.

An introductory speech was made by Mr. J. G. Wainwright, the Treasurer, welcoming Lord Lister, and stating the circumstances which had necessitated some scheme of extension. In 1890 considerable alterations were made in the then existing theatres, but in consequence of the great increase in the number of operations, which rose from 791 in 1890 to 2,810 in 1898, far larger accommodation had become essential if the work of the hospital were to be adequately carried on. Also more beds for children were urgently needed, the inconvenience of one ward only being in use was constantly shown by its enforced closing on account of the recurrence of measles, whooping-cough, &c.

Lord Lister, who was received with much enthusiasm after acknowledging the terms in which Mr. Wainwright had spoken of himself and his work, said he felt it a great honour to be asked to take part in the inauguration of additions to so famous a hospital as St. Thomas's, which, dating from the period of the Norman kings, was now one of the most important institutions of its kind, and had been served by men of the highest distinction in the medical world. Of those still living there was for instance the veteran Sir John Simon, who would have been present that day had age and infirmity permitted; he was for a long time surgeon to St. Thomas's, and his sanitary work was one of the great achievements of the Victorian era. Then, to revert to the past, there was Cheselden, who, in his love for his fellow-men, which was the first of all requirements for the surgeon, in his power of concentration, and in his manipulative skill, exhibited all the qualities necessary for the scientific surgeon. These were samples of the men who had served St. Thomas's, and he felt himself highly honoured at being asked to assist at the opening of additions to an institution with such antecedents. These additions were of a twofold character. First, there were the new theatres for operating; and second, the children's ward. In spite of the statements made in certain quarters —statements which were only pardonable on account of the gross ignorance of those that made them—people, instead of having to be persuaded by their physicians, now came of themselves to hospitals in shoals asking to be treated, and the increased number of operations that was the result necessitated additions to the theatres. These had been most ingeniously planned. The old theatre was divided by a horizontal partition, and the upper of the apartments thus obtained divided vertically to form two theatres, the lower being converted into perhaps the most beautiful children's ward that had ever existed. In the theatres everything had been done that science could suggest to promote cleanliness and purity. But his surgical friends would not misunderstand him when he said that he regarded such elaborate arrangements rather as luxuries than as essentials. His own work had been done in old-fashioned hospitals, and it was not for him to say whether his practice in those hospitals had been satisfactory or not. But a day or two ago he had inspected the new operating theatre at the Western Infirmary, Glasgow, and there, while he saw much that reminded him of what was to be seen at St. Thomas's the ventilation was on the old-fashioned system; there was no attempt to filter the atmosphere, no sterilisation by heat was practised, and the surgeons did not change their garments before entering the operating room. Yet as good results were obtained there as in any other place in the world. He had some jealousy lest students, taught amid such luxuries as were afforded by the new theatres at St Thomas's, might feel discouraged when performing operations in remote country districts or in the homes of the poor, and give up the attempt to get aseptic results. He would suggest that those teaching in St. Thomas's should remind their pupils that good results might be obtained by simpler means. What he had said was perhaps superfluous, but, if so, he would beg his friends to forgive him, for he had not spoken in any spirit of criticism, but in one of warm admiration for the institution in which they were present.

Prayers were then said by the Bishop of Southwark, and some hymns charmingly sung by a choir of probationers, whose clear and well-trained voices made the music echo most effectively down the long corridors. A procession was formed, and Lord Lister was taken in succession, first to the children's ward, and thence to the theatres, in each dedicatory prayers being said.

The theatres are certainly the most up-to-date of their kind in London, and with their adjuncts, anæsthetising, and sterilisation rooms, are very well arranged. The walls of white "marmorite" (a kind of opaque glass) and Keene's cement, white enamelled; the terrazzo floors, glass and iron fittings, and every detail of glass slab and earthenware sink are as perfect as modern science can contrive. The auditorium is of solid white marble, separated from the floor of the theatre by white enamelled rails. Instead of pedal taps, simple taps, fitted with roses, have been provided for the sinks, so that a spray of water is kept running during the whole time operations are in progress, for the cleansing of the surgeon's hands. In the surgeon's room rubber boots and sterilised blouses are kept for the use of the operators and their assistants.

The children's ward, containing sixteen cots, is named the "Lilian" in memory of the daughter of Mr. S. G. Holland, who has defrayed the cost of the tiling. The walls are of specially made tiles, with a green dado, and above a series of picture panels, representing nursery rhymes and stories, charmingly designed by one of Messrs. Doulton's artists. The floor is of polished teak. Kitchen and bathroom are close at hand, with a room for the sisters across the passage, and a small isolation room adjoining.

Both for theatres and ward the Plenum system of ventilation has been adopted, the air being warmed, filtered, and pumped in by rotary fans, and extracted by a system of ducts leading to the open air.

